# Numerical simulation research on thermal insulation performance of composite heat-insulation zone structure in hydrothermal high-temperature mine

**DOI:** 10.1038/s41598-024-64702-4

**Published:** 2024-06-18

**Authors:** Yanhe Li, Zhijun Wan, Zhenzi Yu, Peng Shi, Bo Zhang, Yuan Zhang

**Affiliations:** 1 Pingdingshan Tianan Coal Mining Co., LTD., Pingdingshan, 467009 China; 2 School of Mines, China University of Mining and Technology, Xuzhou, 221116 China; 3 State Key Laboratory of Coking Coal Exploitation and Comprehensive Utilization China Pingmei Shenma Group, Pingdingshan, 467009 China

**Keywords:** Composite heat-insulation zone, Heat-regulating circle, Mine heat damage control, Seepage-heat transfer, Coal, Civil engineering

## Abstract

In hydrothermal high-temperature abnormal mines, the composite heat-insulation zone structure, formed through a combination of guniting and grouting, serves to mitigate heat dissipation from the surrounding rock into the airflow. To comprehensively understand the thermal insulation performance of the composite heat-insulation zone structure, this study employs numerical simulation to analyze the following aspects: the variation in the temperature field within the surrounding rock of the roadway without insulation, the influence of structural parameters of the composite heat-insulation zone on temperature distribution in the surrounding rock of the roadway, and the thermal insulation effectiveness of the composite heat-insulation zone with varying structures. The findings indicate that the temperature distribution within the surrounding rock of the roadway lacking a heat-insulation zone is relatively uniform. However, as ventilation time extends, the heat regulation zone within the surrounding rock gradually extends deeper, ultimately forming an elliptical cooling area. The composite heat-insulation zone structure effectively mitigates heat transfer from deeper surrounding rock to the roadway wall, consequently altering the scope of the roadway's heat regulation zone. Enhancing the thermal insulation performance of the composite heat-insulation zone structure can be achieved by increasing the thickness of the thermal insulation layer, adjusting grouting rate and depth, and reducing the thermal conductivity of insulation materials. The thermal insulation effectiveness of the thermal insulation layer surpasses that of the grouting layer, with its performance primarily influenced by the thermal conductivity of the materials used. Simulation results demonstrate that the composite heat-insulation zone structure reduces the maximum heat flux on the roadway wall from 47.4 to 37.7 W/m^2^, resulting in a 20% reduction in heat transfer from deeper surrounding rock. These findings offer valuable insights for implementing thermal insulation techniques in hydrothermal high-temperature anomaly mines.

## Introduction

Prolonged and intensive global coal mining has resulted in the gradual depletion of shallow coal resources, prompting a shift towards mining in deeper areas of the earth^1–3^. Nevertheless, the consequent issue of heat damage not only impacts the safe and efficient operation of coal mines but also poses risks to miners' physical and mental well-being^4,5^. Therefore, it is a major new challenge for coal mine development to control thermal damage in mines. 

According to incomplete statistics, China's high-temperature mines throughout the 17 provinces (autonomous regions), over 60 mine areas, and 200 mines, involving the capacity is more than 360 Mt^6,7^. These high-temperature mines are mainly located in Henan, Hebei, Anhui, Jiangsu, etc. of China, especially in the coalfield of Pingdingshan, Henan, which is the most prominent. This area has been influenced by special tectonic belts, which have changed the direction of the earth's heat flow, resulting in the phenomenon that local heat gathering occurs in the strata within this area so that the geothermal reservoir of high temperature has been formed^8^. In addition, the area has multiple outcrops of limestone, which together with discontinuous surfaces such as stratigraphic joints and fissures provide channels for surface water transport to the geothermal reservoir, thereby forming confined hot water and aggravating the thermal damage of the mine while there being potential flooding damage^9–11^. Therefore, it is important to study the water-thermal control of hydrothermal high-temperature anomalous mines.

Presently, heat damage control in high-temperature anomalous mines primarily relies on mechanical cooling methods^12–14^, such as underground air-conditioning systems. Research indicates^15,16^ that mechanical cooling methods effectively enhance the underground thermal environment. Nevertheless, the capital investment required for constructing cooling systems and the ongoing operational expenses of cooling units are prohibitively expensive for small and medium-sized enterprise (SME) mines. Additionally, this method does not address the risk of potential flooding damage. So, some scholars have turned their research direction to cooling through thermal insulation of mines and obtained fruitful results. Some of them have studied the effects of thermal insulation layer parameters (thermal conductivity and thickness) and virgin rock temperature on the temperature field of the surrounding rock in the roadway, the heat-regulating circle, the temperature of the roadway wall, and the heat flux in between the roadway wall and the airflow by numerical simulation. For example, Szlazak et al.^17^ analyzed the impact of thermal insulation on the surface of the roof and sidewalls on the reduction in heat transfer from the strata with a high virgin temperature to mine air, by comparing the climate on the roof and sidewalls when they were insulated and without thermal insulation. And pointed out that insulation can reduce heat flow by 75% in the face zone. Gao et al.^18^ explored the effects of thermal insulation layer thickness and thermal conductivity, convective heat transfer coefficient between roadway wall and airflow, and roadway radius on the thermal insulation performance of thermal insulation roadway, and showed that the effects of thermal insulation layer thickness and thermal conductivity on the thermal insulation performance of roadway are greater than the roadway radius. Others have studied thermal insulation materials, mainly exploring the thermal conductivity of different ratios of materials and the effectiveness of on-site thermal insulation. For instance, Xiao et al.^19^ utilized nanoporous superinsulation materials in deep metal mines, demonstrating that these materials effectively inhibit gas heat conduction. Specifically, when the heat source temperature is 200 °C, the coating surface temperature of the thermal insulation material (excluding aerogel) is 100 °C, resulting in a thermal insulation temperature difference of 100 °C. In contrast, when using aerogel thermal insulation coating with a coating surface temperature of only 60 °C, the thermal insulation temperature difference increases to 140 °C. Jiang et al.^20^ developed heat-insulating materials using basalt fibers and high-strength ceramsite combined with cemented materials. The performance indicators corresponding to the optimal comprehensive combination of these heat-insulating materials included a density of 1200 kg/m, thermal conductivity of 0.151 W/(mK), compressive strength of 9.7 MPa, flexural strength of 3.6 MPa, and a water-seepage depth of 25.4 mm.

The majority of the aforementioned studies involve the application of thermal insulation materials sprayed onto the roadway walls to impede heat dissipation from the surrounding rock into the airflow. This approach has been experimentally validated as effective in mitigating thermal damage in mines. Nevertheless, this method presents certain drawbacks for hydrothermal high-temperature anomalous mines. The primary concerns include: (1) Difficulty in achieving a balance between thermal insulation, strength, and cost of mine insulation materials, leading to potential failure of the sprayed insulation materials due to mining activities. (2) Hydrothermal high-temperature anomalous mines often experience significant surrounding rock seepage, with groundwater infiltrating through fissures and facilitating heat migration. This process exacerbates heat damage in the mines during the heating of the surrounding rock. Consequently, some scholars have proposed combining thermal insulation, blocking, and support mechanisms through the use of composite heat-insulation zone structures^21–23^ to manage heat dissipation from the surrounding rock. Nevertheless, the precise impact of each parameter (guniting thickness, grouting range, and grouting depth) on the heat dissipation characteristics of the roadway surrounding rock remains unclear. Therefore, this paper analyzes the variation in the temperature field of the surrounding rock without a heat-insulation zone and investigates the influence of the structural parameters of the composite heat-insulation zone on the temperature field in the surrounding rock by establishing a numerical model. The paper is structured as follows: "Study of basic parameters for thermal damage control in mines" section introduces the study of basic parameters for thermal damage control in mines, "Numerical simulation study of temperature field in the surrounding rock of the composite heat-insulation zone roadway" section presents the numerical simulation study of the temperature field in the surrounding rock of the composite heat-insulation zone roadway, and "Results and discussion" section discusses the results and provides analysis. Finally, several important conclusions are drawn in "Conclusion" section.

## Study of basic parameters for thermal damage control in mines

### Overview of the thermal environment of the mine

Pingdingshan Tianan Coal Joint Stock Company Limited No. 10 Mine is situated in the northeast of Pingdingshan City, approximately 5 km from the city center. According to mine data, the average geothermal gradient of the mine is 3.4 °C/hm, with localized areas reaching up to 4.6 °C/hm. Additionally, carbonate rocks are extensively developed in strata deeper than 1000 m within the mine area, containing a substantial amount of hot water, posing a long-term flooding threat to mine safety. In response, a roadway has been excavated below the gateway at the − 870 m level, and drainage measures have been implemented.

**Figure 1:**
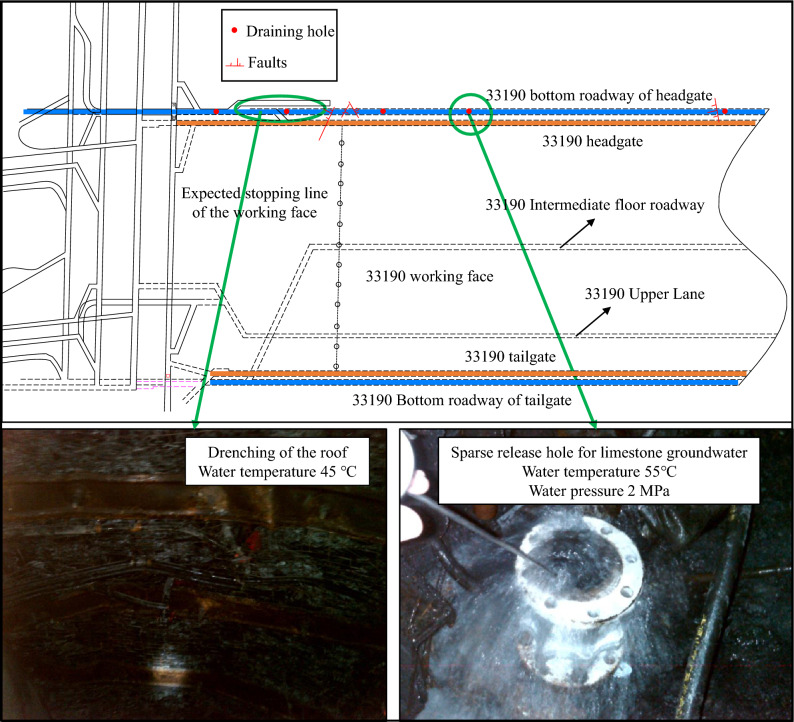
Overview of the bottom tunnel layout of the 33,190 haulage gateway.

An in-depth investigation was conducted on the roadway situated 16–22 m below the floor level of the 33,190 haulage gateway. The roof of the roadway is excavated along the L2 limestone floor, and the dimensions of the roadway measure 4800 mm in width, 3800 mm in height, with a total length extending 1500 m. According to on-site measurements, the average temperature of the roadway wall is recorded at 37 °C, while the wind temperature at the tunneling face registers at 34.2 °C. Concurrently, the water pressure at the draining hole ranges from 1.5 to 2.5 MPa, with a water temperature between 52 and 55 °C. Moreover, local observations reveal instances of roof drenching, reaching temperatures of up to 45 °C, as depicted in Fig. 1.

### Determination of basic physical parameters of rocks

**Figure 2:**
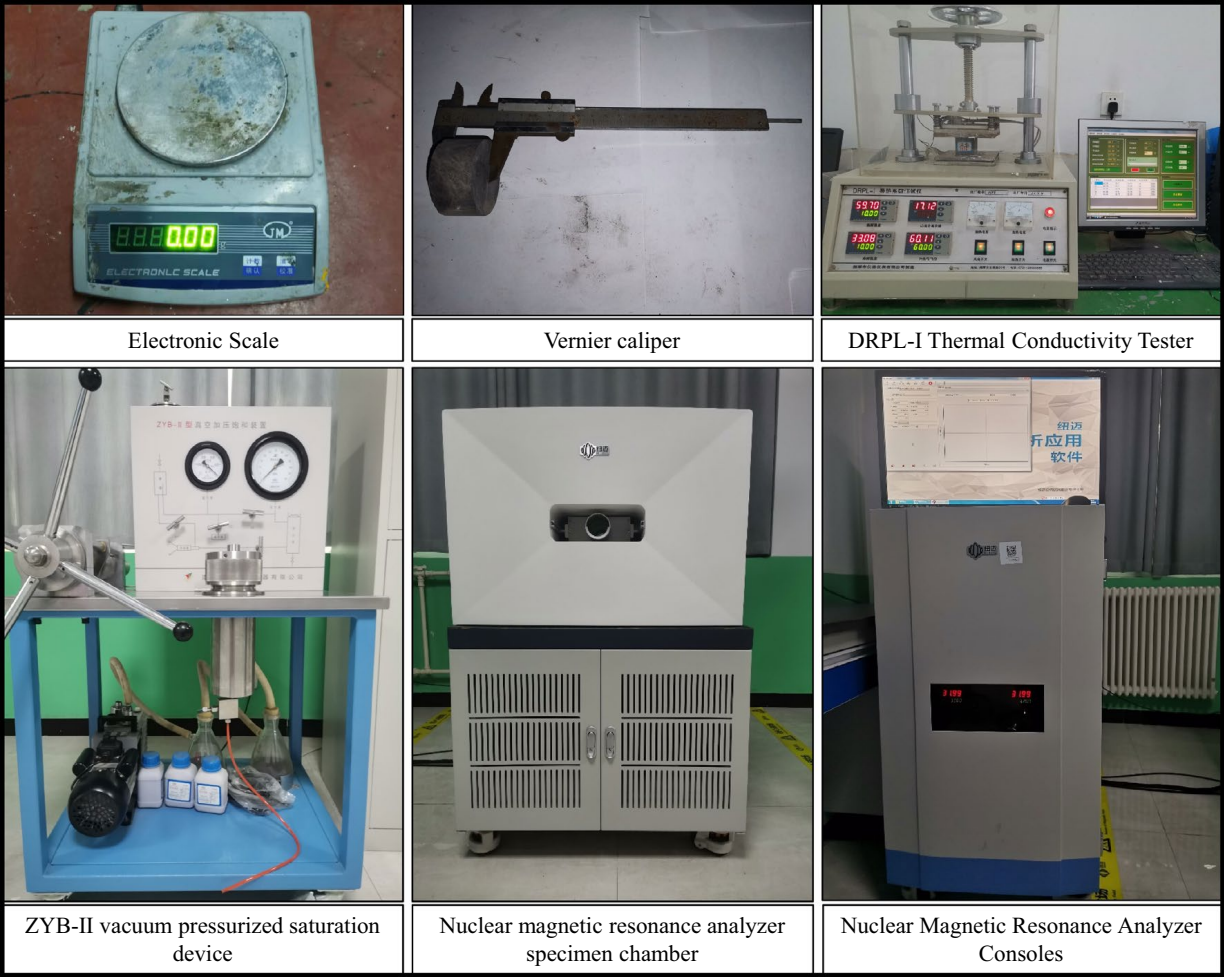
Test instrument.

Rock specimens were extracted from the drill cores of the bottom tunnel at the 33,190 haulage gateway for parameter determination, including density, porosity, and thermal conductivity. The necessary test instruments are depicted in Fig. 2. Density measurements were conducted using a balance and vernier caliper, while porosity was determined using a ZYB-II vacuum pressurized saturation device and NMR analyzer. Thermal conductivity testing was performed using a DRPL-I thermal conductivity tester. The summarized test results are presented in Table 1.

**Table 1:** Test results of specimen base parameters.

Number	Lithology	Density (kg/m^3^)	Porosity	Thermal conductivity (W/(m·°C ))
S_1	Sandy mudstone	2.59	0.13	1.93
S_2	Sandy mudstone	2.57	0.15	1.97
S_3	Sandy mudstone	2.56	0.16	1.98
T_1	Carboniferous limestone	2.71	0.21	2.18
T_2	Carboniferous limestone	2.69	0.26	2.22
T_3	Carboniferous limestone	2.67	0.28	2.38
H_1	Cambrian limestone	2.73	0.28	2.36
H_2	Cambrian limestone	2.72	0.29	2.48
H_3	Cambrian limestone	2.70	0.33	2.51

### Raw rock temperature determination

#### Temperature measurement equipment

**Figure 3:**
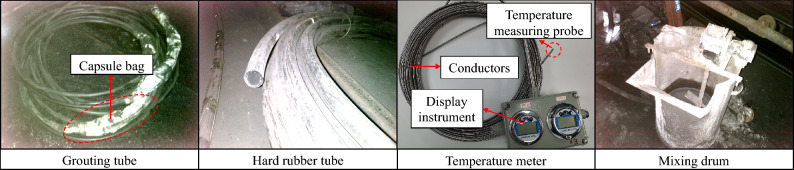
Temperature measurement equipment.

Figure 3 displays the equipment necessary for temperature measurement in the field. The Explosion-proof multi-point in-situ display temperature meter was utilized to measure the temperature of the borehole enclosure, comprising three main components: wire (encased in flexible steel material), temperature measuring probe, and display instrument. A grouting pipe (equipped with bladder bags at both ends) was employed to seal the hole after embedding the temperature sensor, thereby mitigating the influence of groundwater and air on the borehole's temperature field. Additionally, a hard rubber hose facilitated the insertion of the temperature sensor, ensuring precise delivery to the pre-embedded location.

#### Drilling parameters

The on-site temperature measurement target area was arranged in the bottom tunnel of the 33,190 haulage gateway, with a distance of 350 m and 600 m from the mouth of the solid coal side of the tunnel entrance. Among them, the drilling depth is 25 m, the diameter is 94 mm, the dip angle is 2 ~ 5° upward, and the azimuth angle is 0°, as shown in Table 2.

**Table 2:** Field temperature measurement drilling parameters.

Station location (m)	Drilling depth (m)	Drilling diameter (mm)	Drilling inclination (°)	Azimuthal angle (°)	Number of sensors	Sensors location (m)
350	25	94	2–5	0	4	5, 10, 20, 25
600	25	94	2–5	0	2	15, 25

#### Temperature sensors pre-buried process

**Figure 4:**
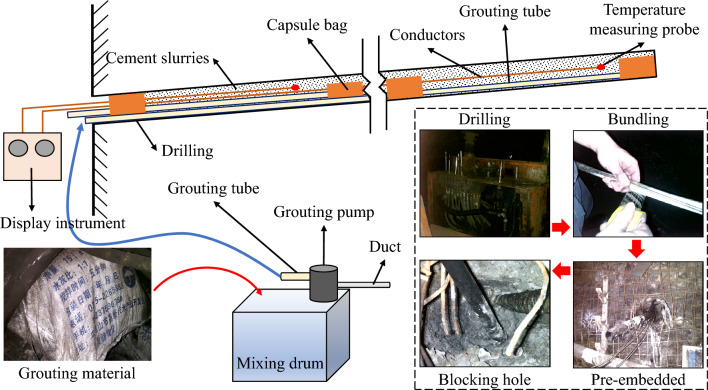
Temperature sensors pre-embedded process flow on site.

The site temperature measurement process is depicted in Fig. 4. Initially, drilling was conducted at the temperature measurement point according to the drilling parameters. During drilling, the grouting pipe, temperature measurement wire, and hard rubber tube were secured together using transparent adhesive tape. Following the completion of drilling, the bundled hard rubber tube was inserted into the borehole and gradually extended to the desired depth until it reached the pre-buried position of the temperature measurement probe. Subsequently, KFL-1 hole sealing grout was mixed with water in a 1:1 ratio and thoroughly mixed. The pneumatic grouting pump was then utilized to inject the slurry into the hole, ceasing the grouting process when the slurry began to flow out of the hole. Temperature measurement commenced after sealing the hole for 24 h (approximately the thermal equilibrium time) to mitigate the influence of the slurry on the hole wall temperature.

#### Temperature measurement results and analysis

**Figure 5:**
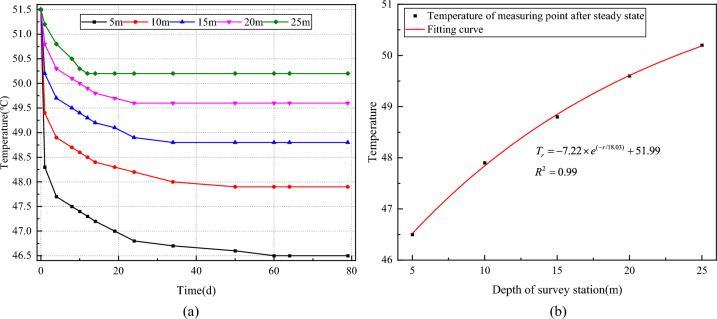
Temperature measurement by drilling. (**a**) Temprature measurement result. (**b**) Temprature data fitting.

The data recording was performed on-site by recording the rock temperature data of the deep part of the temperature measurement hole (25 m) as *T*_*a*_ and *T*_*b*_, and then the value of (*T*_*a*_ − *T*_*b*_)/2 was used as the compensation value of the measured rock temperature of the temperature measurement hole, and the results are shown in Fig. 5a.

Figure 5a illustrates that, on a spatial scale, the temperature gradually increases with the deepening of temperature measurement points into the surrounding rock, accompanied by a decrease in the temperature difference between adjacent measurement points. This phenomenon arises from the initial heat exchange between airflow in the ventilation roadway and the roadway wall, resulting in a temperature reduction, thereby perturbing the surrounding rock temperature field. However, this perturbation effect diminishes as the depth of the surrounding rock increases.

On a temporal scale, the temperature values at each depth gradually decrease with the extension of ventilation time. Notably, there are significant variations in temperature changes at different depths. Specifically, temperature values at shallow measurement points exhibit rapid changes over time, while temperature values at deep measurement points experience a minor initial decrease followed by stabilization. This discrepancy is attributed to the higher initial temperature of well fluid during drilling, causing the initial temperature measurement to reflect the well fluid temperature. Subsequently, after reaching thermal equilibrium, the temperature of deep measurement points remains constant, while the temperature of shallow surrounding rock gradually decreases due to airflow until reaching a new equilibrium state.

The temperature measurement results were further analyzed to determine the raw rock temperature of the roadway. The literature^9^ shows that the surrounding rock causeless temperature and the causeless radius approximation conform to an exponential relationship, which can be expressed as $T = - a \times e^{( - r/b)} + c$ (T is the surrounding rock temperature within the heat-regulating circle, °C; r is the radius of the heat-regulating circle, m; a, b, c are the fitted correlation positive coefficients). Based on this, the temperature measurement results were fitted and the results are shown in Fig. 5b.

It can be seen from Fig. 5b that the fitted relationship of depth-temperature of the surrounding rock at the roadway is: *T*_*r*_ = − 7.22 × e^(−*r*/18.03)^ + 51.99 °C. Meanwhile, the curve fitted well (*R*^2^ = 0.99) and can be used to predict the raw rock temperature of the roadway. At this time, the drilling depth is *r* ≥ 37.5 m and *T*_(*r* = 37.5)_ = 51.1 °C. It can be known that the raw rock temperature of the roadway is 51.1 °C.

## Numerical simulation study of temperature field in the surrounding rock of the composite heat-insulation zone roadway

### Structural model of composite heat-insulation zone

**Figure 6:**
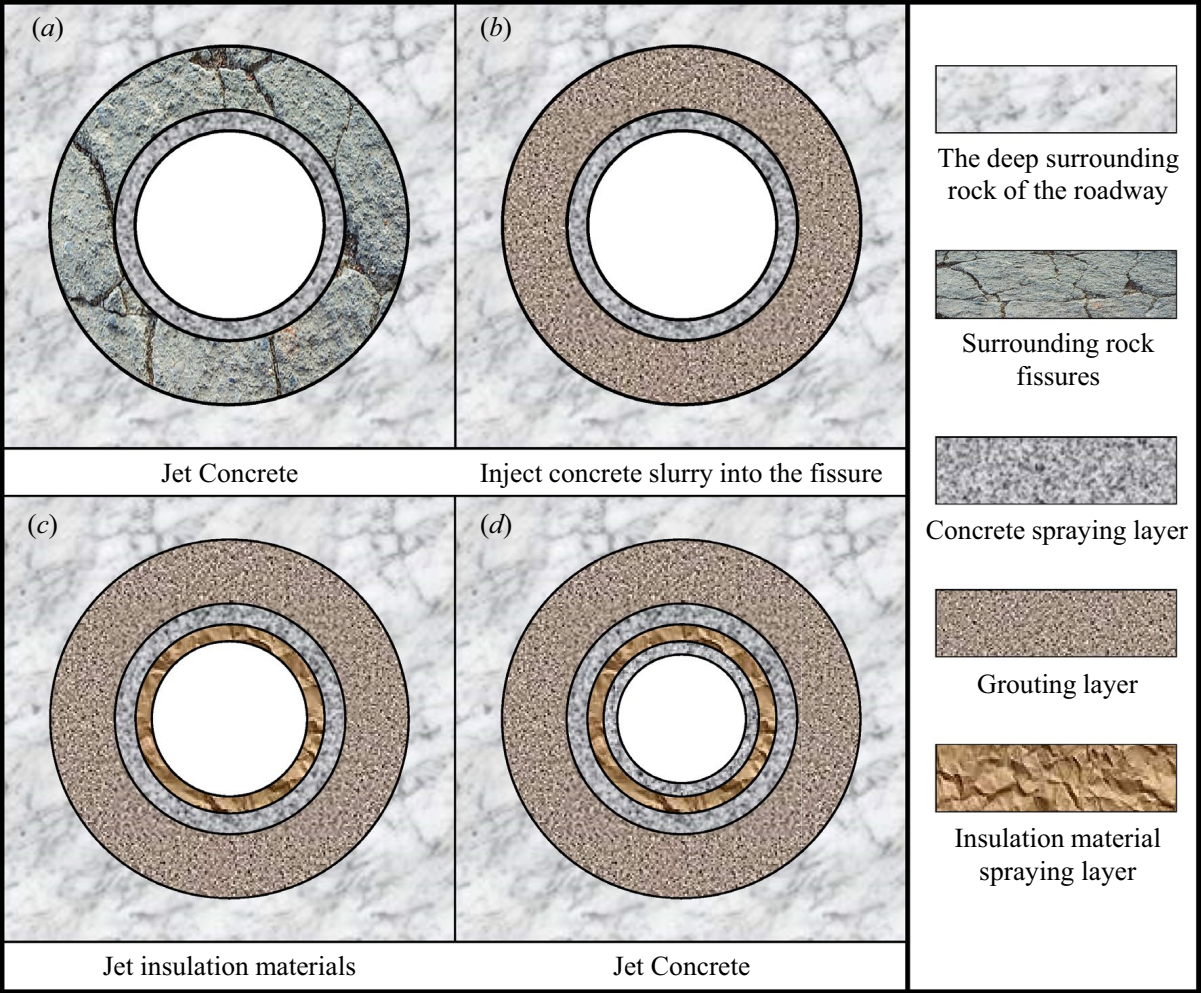
Flow chart of the composite heat-insulation zone structure and implementation.

The literature^22,23^ presents the model of the composite heat-insulation zone structure, depicted in Fig. 6. Following the excavation of the roadway, a layer of concrete is sprayed onto its surface. Subsequently, cement slurry is injected into the surrounding rock relaxation zone through grouted rock bolts or grouted anchor cables, forming an inner heat-insulation zone structure to mitigate heat migration towards the roadway during geothermal water seepage. Guniting is employed to prevent slurry outflow during grouting. Upon the formation of the inner heat-insulation zone structure, a layer of material insulation is sprayed onto the roadway surface to minimize heat transfer from the surrounding rock. Finally, a layer of concrete is sprayed onto the roadway to ensure structural stability during coal extraction.

### Model building

**Figure 7:**
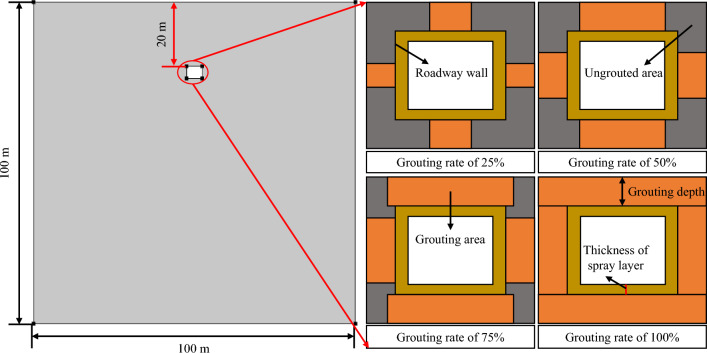
Numerical model of the temperature field in the surrounding rock of the composite heat-insulation zone roadway.

The numerical model of the temperature field of the surrounding rock in the composite heat insulation zone roadway was constructed based on the mine geological conditions and the structural characteristics of the composite heat insulation zone using the COMSOL Multiphysics finite element numerical simulation software, as depicted in Fig. 7. The model is represented as a square with a side length of 100 m. The dimensions of the roadway are specified as width × height = 4.8 m × 3.8 m, with the roadway roof positioned 20 m from the upper boundary. Additionally, the grouting area is delineated as a rectangular ring formed by the roadway wall and its grouting depth range. The rectangular ring is further divided into four rectangles, with the ratio of the grouting area to the total area within each rectangle defined as the grouting rate.

### Model parameters and boundary conditions

#### Model parameters

The simulation parameters primarily encompass the fundamental physical characteristics of the surrounding rock, guniting, and grouting materials, as well as the parameters for the composite heat-insulation zone structure. For the simulation, the rock's physical parameters for different layers were configured using segmental functions, while the thermal properties of each parameter were determined based on the test results in "Determination of basic physical parameters of rocks" section. A comprehensive overview of these parameters is provided in Table 3.

**Table 3:** Model parameters.

Name	Rock character	Thickness [m]	Density [kg/m^3^]	Porosity	Permeability [m^2^]	Specific heat capacity [J/(kg·K)]	Thermal conductivity [W/(m·K)]
Surrounding rock	Sandy mudstone	20	2590	0.15	10^−18^	930	1.96
	Carboniferous limestone	17	2710	0.25	10^−17^	850	2.26
	Sandy mudstone	20	2570	0.15	10^−18^	930	1.96
	Carboniferous limestone	16	2670	0.25	10^−17^	850	2.26
	Bauxitic mudstone	7	2560	0.1	10^−18^	910	1.85
	Cambrian limestone	20	2720	0.3	10^−16^	980	2.45
Concrete guniting layer	–	0.8	1800	0.2	10^−16^	850	1.5
Insulation layer	–	–	1350	0.3	10^−15^	500	–
Grouting layer	–	–	2200	0.1	10^−19^	600	1.6

All physical properties of the grout layer are the equivalent physical properties of the rock within the grouting area.

#### Boundary conditions

The numerical model developed in this study addresses the problem of seepage flow and heat transfer in porous media. Simulations conducted using COMSOL Multiphysics necessitated the implementation of two physical field interfaces: Darcy's law and heat transfer in porous media. Model boundary conditions were primarily established based on on-site test results and mine hydrogeological data. Notably, the model boundary temperature was determined using the average geothermal gradient (3.4 °C/hm) and the geothermal test results from the floor of the 33,190 haulage gateway. The upper boundary temperature of the model was set to 51.1–3.4 × 21.9/100 °C (the distance from the floor of the 33,190 haulage gateway to the model upper boundary is 21.9 m), while the lower boundary temperature was set to 51.1 + 3.4 × 78.1/100 °C (the distance from the floor of the 33,190 haulage gateway to the model lower boundary is 78.1 m). Additionally, the left and right boundaries of the model were set to 51.1–3.4 × y/100 °C. Furthermore, the inner boundary of the model was configured for internal forced convection heat transfer, with the relative atmospheric pressure set to 4000 Pa, wind speed set to 0.7 m/s, and wind temperature set to 30 °C. The water pressure reference pumping hole pressure at the lower boundary of the model was set to 2.0 MPa. Detailed boundary conditions are provided in Tables 4 and 5.

**Table 4:** Temperature boundary conditions.

Upper boundary	Lower boundary	Left and right boundaries	Inner boundary
51.1–3.4 × 21.9/100 °C	51.1 + 3.4 × 78.1/100 °C	51.1–3.4 × y/100 °C	*P*_A_ = 4000 Pa
			*v* = 0.7 m/s
			*T*_w_ = 30 °C

*P*_*A*_ is the absolute atmospheric pressure in the roadway, *v* is the airflow velocity, and *T*_*w*_ is the airflow temperature.

**Table 5:** Seepage boundary conditions.

Upper boundary	Lower boundary	Left and right boundaries	Inner boundary
0.1 MPa	2.0 MPa	No flow	0 MPa

### Program design

The experimental design primarily focuses on varying parameters such as grouting depth, grouting rate, insulation thickness, and thermal conductivity of insulation materials, each with four levels, as detailed in Table 6. Group I serves as the basic control group, simulating the temperature field variation of the surrounding rock without the heat-resistant zone structure. Group II investigates the influence of insulation layer thickness on heat dissipation from the surrounding rock wall to the airflow. Group III examines the impact of the thermal conductivity of insulation materials on heat dissipation from the surrounding rock to the airflow. Group IV evaluates the effect of grouting range on heat dissipation from the surrounding rock to the airflow. Finally, Group V explores the influence of grouting depth on heat dissipation from the surrounding rock to the airflow.

**Table 6:** Numerical simulation scheme.

Program	Grouting depth [m]	Grouting range [%]	Thickness of insulation layer [mm]	Thermal conductivity of insulation materials [W/(m·K)]
1	0	0	0	0.4
2	0	0	40/80/120/160	0.4
3	0	0	160	0.1/0.2/0.3
4	1	25/50/75/100	160	0.1
5	2/3/4	100	160	0.1

## Results and discussion

### Model validation

**Figure 8:**
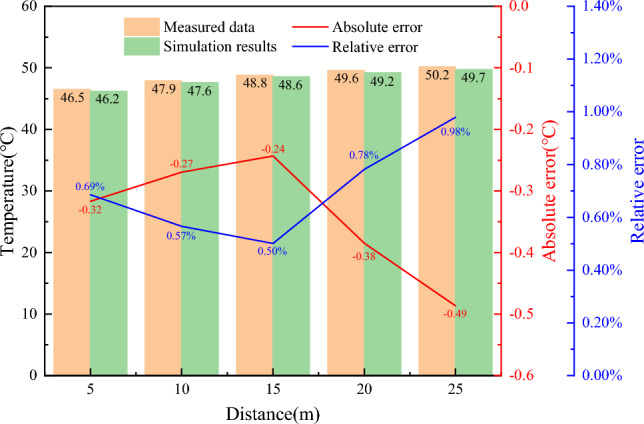
Result validation.

To validate the rationality of the model, the temperature data of the surrounding rock after the steady state of the model roadway waistline at depths of 5 m, 10 m, 15 m, 20 m, and 25 m were obtained, and then compared with the measured results in "Temperature measurement results and analysis" section, and verified the results of the numerical simulation taking the absolute error and the relative error as the characteristic parameter, the result is shown in Fig. 8.

It can be seen from Fig. 8 that the variation law of the surrounding rock temperature with depth after the steady state is almost consistent with the measured results. The error of the surrounding rock 20 m deeper than the shallow part is larger, more than 0.38 °C. However, the overall simulation error is relatively small, the average error is 0.34 °C, the minimum error is 0.24 °C, and the maximum error is 0.49 °C. Meanwhile, the simulated relative error is small as it varies between 0.50 and 0.98%. This shows that the simulation results are reliable and the model can be used to study the thermal insulation performance of the composite heat-insulation zone.

### Variation law of the temperature field of the roadway surrounding rock

**Figure 9:**
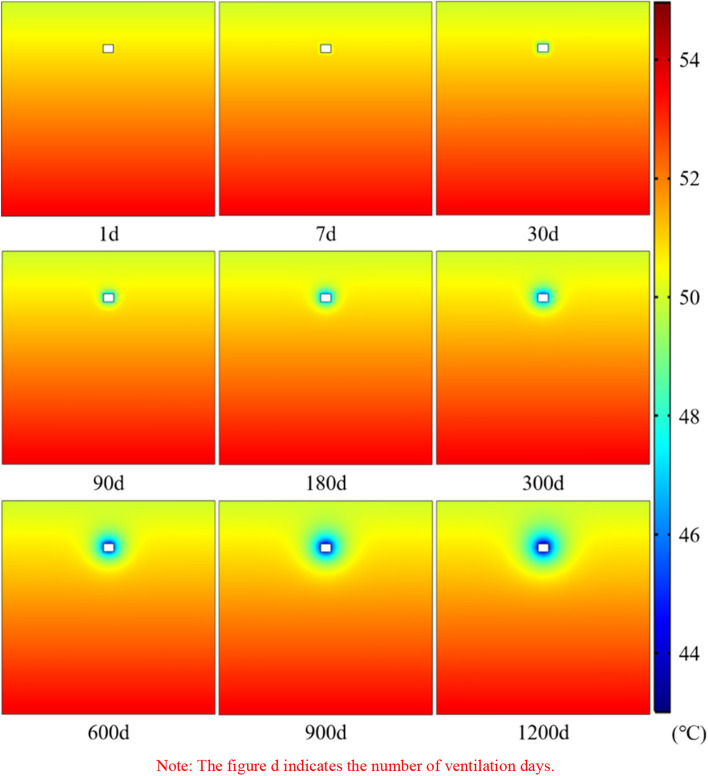
Diagram of the temperature field of the roadway surrounding rock with time.

Figure 9 shows the variations cloud atlas of the temperature field of the roadway surrounding rock with time. It can be seen that the distribution in the temperature field of the roadway surrounding rock is relatively uniform. The surrounding rock continuously heat dissipation to the airflow with the extension of ventilation time, resulting in the temperature of the roadway wall gradually decreasing, the cooling range gradually extending to the deeper area, finally forming an elliptical cooling area. Meanwhile, the temperature difference between the roadway wall and the airflow is larger in the ventilation prophase, and the heat exchange efficiency between the both is faster, which leads to the more obvious temperature drop phenomenon of the roadway surrounding rock.

### Influence of composite heat-insulation zone structural parameters on the temperature field of the surrounding rock

Analyze the influence of each parameter on the temperature field of the surrounding rock by studying the variation in structural parameters of the composite thermal insulation zone on the roadway wall temperature and the radius of the heat-regulating circle. The boundary of the heat-regulating circle is determined by the principle of ∂T/∂r ≤ 0.05 and ∂T/∂t ≤ 0.05 (where T represents temperature, r is the distance of the calculated point in the surrounding rock to the roadway wall, and t is time). Subsequently, the temperature of the surrounding rock along the measuring line (with an interval of 0.1 m) was determined by placing the measuring line on the left waistline of the model roadway, and the radius of the heat-regulating circle for different schemes was calculated based on these principles.

#### Influence of insulation thickness on the temperature field of the surrounding rock

**Figure 10:**
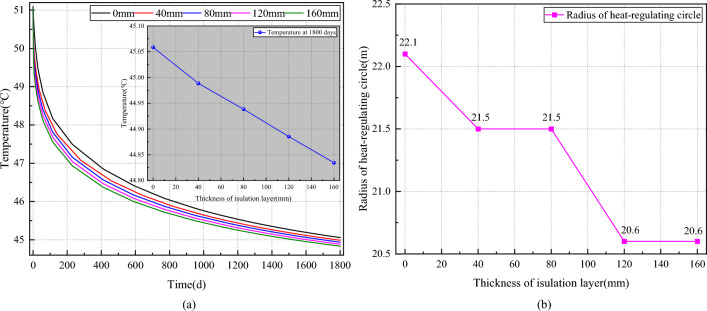
Effect of insulation thickness on the temperature field of the roadway surrounding rock. (**a**) Varation of curve temperature on wall of roadway with thickness of insulation layer. (**b**) Varation of curve radius of heat regulating circle with thickness of insulation layer.

Figure 10 illustrates the impact of insulation thickness on the temperature distribution in the surrounding rock of the roadway. In Fig. 10a, it is observed that the roadway wall temperature decreases exponentially over time. After 1800 days of ventilation, the roadway wall temperature slightly decreases with increasing insulation thickness, measuring respectively at 45.06 °C, 44.99 °C, 44.94 °C, 44.89 °C, and 44.83 °C. Compared to the roadway wall temperature without the insulation layer, these temperatures represent reductions of 0.16%, 0.27%, 0.38%, and 0.51% respectively. Figure 10b depicts the stepwise reduction in the radius of the heat-regulating circle of the roadway as insulation thickness increases, measuring at 22.1 m, 21.5 m, 21.5 m, 20.6 m, and 20.6 m, respectively. Compared to the radius of the heat-regulating circle of the roadway without insulation, these values represent reductions of 2.71%, 2.71%, 6.79%, and 6.79%, respectively.

Summing up, the addition of a thermal insulation layer on the roadway wall effectively reduces the intensity of heat transfer from the deep raw rock to the roadway wall, consequently decreasing heat accumulation on the wall. Consequently, as the insulation layer thickness increases, the airflow cools the roadway wall more rapidly, resulting in a decrease in its temperature. However, the temporal trend of the roadway wall remains unaffected by the construction of the insulation layer. Moreover, while the overall tendency of the heat-regulating circle radius decreases with increasing insulation layer thickness, there are certain thickness intervals where the heat-regulating circle radius remains unchanged. This phenomenon occurs due to reduced airflow disturbance to the temperature field of the surrounding rock within these intervals.

#### Influence of insulation material thermal conductivity on temperature field of the surrounding rock

**Figure 11:**
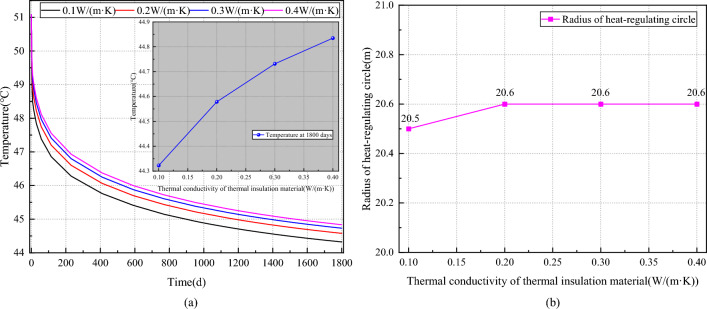
Effect of heat conductivity of insulation materials on the temperature field of the roadway surrounding rock. (**a**) Varation of curve temperature on wall of roadway with thickness of insulation layer. (**b**) Varation of curve radius of heat regulating circle with thermal conductivity of insulation layer.

In Fig. 11a, it is observed that the roadway wall temperature decreases exponentially with time under the influence of the heat conductivity of the insulation layer. After 1800 days of ventilation, the roadway wall temperature gradually decreases with decreasing heat conductivity: 44.83 °C, 44.73 °C, 44.58 °C, and 44.32 °C. Compared to the insulation layer with a heat conductivity of 0.4 W/(m·K), the temperatures of the roadway walls decrease by 0.22%, 0.56%, and 1.14%, respectively. Figure 11b reveals that the heat-regulating circle radius of the roadway exhibits minimal variation with the heat conductivity of the insulation layer. When the heat conductivity decreases from 0.4 W/(m·K) to 0.2 W/(m·K), the heat-regulating circle radius remains unchanged at 20.6 m. Upon further reduction of the heat conductivity to 0.1 W/(m·K), the heat-regulating circle radius decreases to 20.5 m.

Summarizing, the heat conductivity of the thermal insulation layer exerts a greater impact on the roadway wall temperature compared to its thickness. As the heat conductivity of the thermal insulation layer decreases, the thermal insulation effect becomes more pronounced. However, this parameter has a lesser effect on the heat-regulating circle of the roadway surrounding rock. Therefore, prioritizing the reduction of heat conductivity in material selection is crucial in the design of roadway insulation.

#### Influence of grouting rate on the temperature field of the surrounding rock

**Figure 12:**
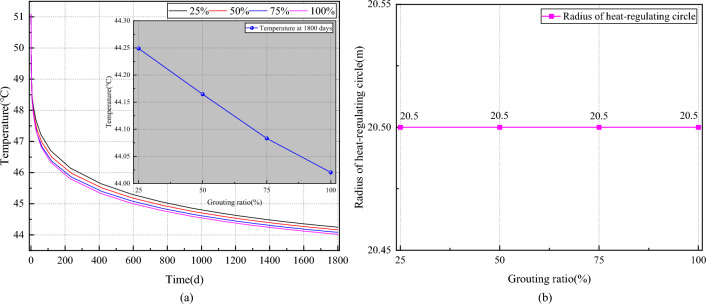
Effect of grouting rate on the temperature field of the roadway surrounding rock. (**a**) Varation of curve temperature on wall of roadway with grouting ratio. (**b**) Varation of curve radius of heat regulating circle with grouting ratio.

Figure 12 shows the effect of the grouting rate on the temperature field of the roadway surrounding rock. It is shown in Fig. 12a that there is no change in the trend of the roadway wall temperature with time for the grouting rate. At ventilation 1800 days, the roadway wall temperature gradually decreases with the increase of grouting rate: 44.25 °C, 44.16 °C, 44.08 °C, and 44.02 °C, respectively. Compared with the grouting rate of 25%, the temperatures of the roadway walls are reduced by 0.19%, 0.37%, and 0.52%, respectively. From Fig. 12b, it is shown that the heat-regulating circle radius of the roadway is always maintained at 20.5 m with the increase of the grouting rate.

In summary, the implementation of a grouting layer in the surrounding rock of the roadway effectively impedes the seepage of geothermal water, thereby reducing thermal migration within the rock mass. Consequently, the thermal insulation effect becomes increasingly pronounced with greater grouting depth. However, the temperature of the roadway wall exhibited a modest decrease of only 0.23 °C when the grouting rate increased from 25 to 100%. This indicates that the influence of the grouting rate on the temperature field of the roadway surrounding rock is minimal when the grouting depth is 1 m. This phenomenon arises because the grouting layer primarily affects the permeability of fissures, whereas the percolation medium of the surrounding rock in this study consists solely of pores. Hence, it suffices to seal the principal water-conducting fissures rather than the entire cross-section of the roadway when designing the composite heat-insulation zone structure.

#### Influence of grouting depth on the temperature field of the surrounding rock

**Figure 13:**
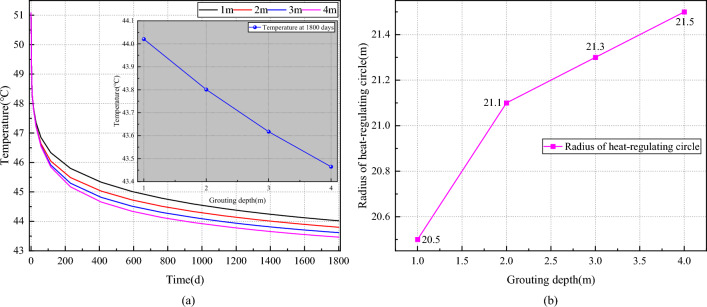
Effect of grouting depth on the temperature field of the roadway surrounding rock. (**a**) Varation of curve temperature on wall of roadway with grouting depth. (**b**) Varation of curve radius of heat regulating circle with grouting depth.

Figure 13 illustrates the impact of grouting depth on the temperature field of the roadway surrounding rock. As depicted in Fig. 13a, the roadway wall temperature decreases exponentially with increasing grouting depth over time. At ventilation for 1800 days, the roadway wall temperature gradually decreases with increasing grouting depth, measuring 44.02 °C, 43.80 °C, 43.62 °C, and 43.46 °C, respectively. Compared with a grouting depth of 1 m, the temperature decreases by 0.50%, 0.92%, and 1.26%, respectively. In Fig. 13b, it is observed that the heat-regulating circle radius of the roadway surrounding rock gradually increases with grouting depth, albeit at a diminishing rate. The respective heat-regulating circle radii are 20.5 m, 21.1 m, 21.3 m, and 21.5 m with increasing grouting depth. Compared with a grouting depth of 1 m, this represents an increase of 2.93%, 3.90%, and 4.88%, respectively. The construction of the grouting layer at the roadway wall effectively reduces the seepage strength of geothermal water in the surrounding rock, thereby mitigating thermal migration from the surrounding rock to the roadway wall. Consequently, this leads to the expansion of the perturbation range of the airflow to the temperature field of the surrounding rock within the roadway.

In summary, it is evident that constructing a grouting layer at the roadway wall can mitigate the heat exchange between the surrounding rock of the grouting layer and the geothermal water. Therefore, when establishing the grouting layer in hydrothermal high-temperature mines, it is crucial to consider the depth of heat exchange between the geothermal water and the grouting layer. The determination of on-site grouting depth should be based on this consideration.

### Analysis of thermal insulation performance of composite heat-insulation zone structure

**Figure 14:**
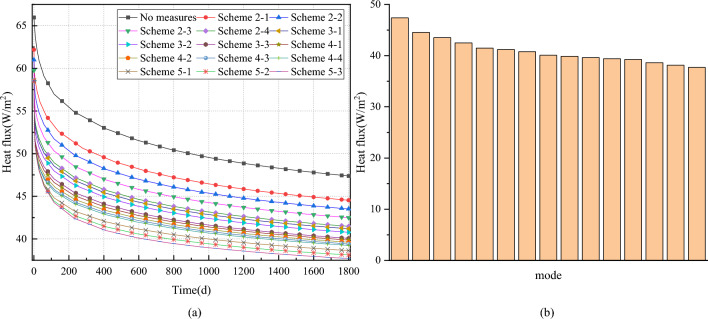
Variation curve of heat flux at the roadway wall of composite heat-insulation zone. (**a**) Varation of curve heat flux of roadway with time under different working conditions. (**b**) Varation of curve heat flux of roadway wall under different working conditions at 1800d.

The heat flux between the surrounding rock and the airflow serves as a crucial indicator for assessing the thermal insulation efficacy of the roadway, calculated by the product of the internal forced convection heat transfer coefficient and the roadway wall area. Figure 14 illustrates the variation curve of heat flux at the roadway wall of the composite heat-insulation zone.

Figure 14 illustrates the variation curve of heat flux at the roadway wall of the composite heat-insulation zone. On a temporal scale, the heat flux at the roadway wall of the composite heat-insulation zone exhibits an exponential decrease. This decline primarily arises from the emergence of a temperature difference between the airflow and the roadway wall post-ventilation, leading to a gradual reduction in the temperature of the roadway wall. Consequently, the thermal intensity of the roadway wall diminishes with the extension of ventilation time. Regarding spatial considerations, the heat flux progressively diminishes as the composite heat-insulation zone structure is established adjacent to the roadway wall. By ventilation at 1800 days, the heat flux at the roadway wall decreased from 47.4 to 37.7 W/m^2^, highlighting that the complete composite heat-insulation zone can block at least 20% of the heat emanating from the deep surrounding rock. Moreover, the parameters of the thermal insulation layer exert a more significant influence on the heat dissipation of the roadway wall compared to those of the grouting layer.

Summing up, it can be seen that the setting of the grouting layer is mainly to block the heat migration of geothermal water to the roadway wall through the fissure, and the setting of the thermal insulation layer is mainly to improve the thermal insulation effect of the composite heat-insulation zone. During the structural design of the composite heat-insulation zone, the seepage field and temperature field distribution law of the roadway surrounding rock should be considered comprehensively, and the most appropriate structural parameters of the composite heat-insulation zone were determined by analyzing the influence of the thermal insulation layer and the grouting layer on the heat conduction and heat convection of the surrounding rock.

## Conclusion

This study conducted tests on the fundamental physical parameters of the rock beneath the roadway and measured its raw temperature. A numerical model was developed to analyze the temperature distribution in the composite heat-insulation zone surrounding the roadway. Additionally, the temperature variation in the non-heat-insulation zone was examined. Furthermore, the impact of thermal insulation layer and grouting layer parameters on the roadway's temperature field and the thermal insulation efficacy of the composite heat-insulation zone structure was investigated. The subsequent sections yielded valuable findings.

1. Roadway temperature measurements indicate that shallow measurement points exhibit more rapid temperature fluctuations compared to deeper points over time. Deep measurement points experience an initial slight decrease in temperature, followed by stability. The initial rock temperature of the roadway was 51.7 °C.
2. Without a heat-insulation zone, the temperature distribution in the surrounding rock of the roadway remains relatively uniform. However, as ventilation time increases, a heat-regulation circle forms, gradually extending into deeper areas and eventually shaping into an elliptical cooling zone.
3. The composite heat-insulation zone structure effectively mitigates heat transfer from deeper surrounding rock to the roadway wall, thereby altering the extent of the roadway's heat-regulation circle.
4. Enhancing the thickness of the thermal insulation layer, grouting rate, and grouting depth, while decreasing the thermal conductivity of insulation materials, can enhance the thermal insulation efficacy of the composite heat-insulation zone structure.
5. The thermal insulation layer demonstrates superior performance compared to the grouting layer, with its effectiveness primarily influenced by the thermal conductivity of its materials.
6. The simulated composite heat-insulation zone structure reduces the maximum heat flux of the roadway wall from 47.4 to 37.7 W/m^2^, resulting in a 20% decrease in heat transfer from the deeper surrounding rock.

### Supplementary Information

Supplementary Information.

## Data Availability

All data generated or analysed during this study are included in this published article and its supplementary information files.
